# The Preferences of Patients With Cancer Regarding Apps to Help Meet Their Illness-Related Information Needs: Qualitative Interview Study

**DOI:** 10.2196/14187

**Published:** 2019-07-31

**Authors:** Rebecca Richards, Paul Kinnersley, Kate Brain, John Staffurth, Fiona Wood

**Affiliations:** 1 University of Westminster London United Kingdom; 2 Centre for Medical Education Cardiff University Cardiff United Kingdom; 3 Division of Population Medicine Cardiff University Cardiff United Kingdom; 4 Section of Oncology Palliative Care Medicine Cardiff University Cardiff United Kingdom

**Keywords:** education, medical, medical information exchange, smartphone, mobile apps

## Abstract

**Background:**

The shift from inpatient to outpatient and community cancer care means that more patients with cancer need to manage their condition at home, without the direct supervision of their clinician. Subsequently, research has reported that many patients with cancer have unmet information needs during their illness. Mobile devices, such as mobile phones and tablet computers, provide an opportunity to deliver information to patients remotely. Before designing an app intervention to help patients with cancer to meet their information needs, in-depth qualitative research is required to gain an understanding of the views of the target users.

**Objective:**

We aimed to develop an app intervention to help patients meet their illness-related information needs in noninpatient settings. This study explored the information needs of patients with cancer and their preferences for an app and desired app features. Specifically, the perceived acceptability of an app, desired app features, and the potential benefits and disadvantages of, and barriers to, an app were explored.

**Methods:**

Qualitative, one-on-one semistructured interviews were conducted with patients with urological, colorectal, breast, or gynecological cancers (N=23) across two hospitals in South Wales. Interviews were audio-taped, transcribed, and analyzed using a thematic analysis.

**Results:**

Findings indicated that barriers to information exchange and understanding in consultations, and identification of reliable information sources between consultations, appeared to contribute to patients’ unmet information needs. Consequently, app feature suggestions included a question prompt list, a glossary of cancer terms, a resources feature, and a contacts feature. Anticipated benefits of this type of app included a more informed patient, improved quality of life, decreased anxiety, and increased confidence to participate in their care. The anticipated barriers to app use are likely to be temporary or can be minimized with regard to these findings during app development and implementation.

**Conclusions:**

This study highlights the desire of patients with cancer for an app intervention to help them meet their information needs during and between consultations with their clinicians. This study also highlights the anticipated acceptability and benefits of this type of intervention; however, further research is warranted.

## Introduction

Survival rates for patients with cancer in the United Kingdom have doubled in the past 40 years, so for many patients, cancer is a chronic condition that they live with for many years [1]. Consequently, there has been a shift from inpatient to outpatient and community cancer care, where patients manage their condition at home, with less regular supervision by clinicians. This requires patients to take a more active role in their treatment and survivorship. To do this and to cope with and manage these changes in daily life, patients require relevant information [2]. Consequently, government initiatives and National Health Service (NHS) plans, such as the national cancer strategy, have highlighted information provision as one of their key priorities for 2015 to 2020 [3].

Studies suggest that patients generally want information on the extent of the disease, prognosis, available treatments, side effects of treatment, self-care, and return to normal life [4-6]. Other, less urgent, information needs include the impact of the illness on social activities, family and friends, mental well-being, sexual activity, and the risk of others getting cancer [4-6]. In this paper, the term “illness-related information needs” includes information related to the disease itself, treatment, psychological support services, and practical support.

Although many people with cancer want as much information as possible about their condition [7], studies across the United States and Europe have reported high rates of unmet information needs [4,8]. Apart from limiting patients’ ability to participate in their care, unmet information needs are associated with a lower quality of life, loss of control over one’s life, increased anxiety and depression, and dissatisfaction with care [9-12].

The introduction of smart technology, including smartphones and tablet computers, has provided a new platform for delivering information-based interventions to patients. Surveys demonstrate the increasing popularity of digital information resources due to increased access to devices and availability of information websites and apps [13-16]. Cancer outpatients in France reported that half of them had used websites and a quarter of them had used apps to search for health information [14]. The acceptance of new information resources for patients is expanding as expected in line with generational trends and over time [15,16]. The UK government has encouraged the integration of interventions delivered by mobile technology into traditional health care services [17]. Furthermore, key reviews, such as the NHS Five Year Forward review [18] and the Wachter review [19], have highlighted the importance of, and urgent push for, digitization in the NHS to provide a high level of health care at an affordable cost.

A recent systematic review has identified that the current mobile interventions for patients with cancer are limited to mainly helping patients with their treatment or symptom-related information needs [20]. More comprehensive interventions are required for patients managing their condition in noninpatient settings.

This study is part of a series documenting the systematic development of an app to help patients with cancer to meet their illness-related information needs [20]. The primary aim of this study was to explore the potential value of an app for patients with cancer and to establish the type of app required. This included exploration of preferences for an app and its features and the potential benefits and disadvantages of, and barriers to, this type of intervention, as well as the types of patients and the time at which they might find an app most useful. This information will help anticipate the potential uptake of the intervention and its possible outcomes, including potential benefits and disadvantages. Ultimately, this process enables development of interventions that are more relevant and engaging for users and helps to circumvent or minimize common issues in digital intervention research, such as low uptake and adherence [21].

## Methods

### Overview

Semistructured interviews were conducted with patients with cancer in their homes between November 2014 and February 2015. NHS ethical approval and R&D approval was granted (14/WA/0066). Semistructured interviews were chosen as they enable a more personal and in-depth response from individuals compared with quantitative methods [22]. This method also allows participants the freedom to bring up other relevant issues [23].

### Participants

Maximum variation sampling was used to enable divergent views to emerge [24]. Participants were recruited from colorectal, urological, breast, and gynecological cancer clinics within the University Hospital Wales and Velindre hospital in South Wales, United Kingdom. These 4 cancer types were chosen to have a variety of some of the most common cancers in the participant sample [25]. A decision was made to include a varied sample of patients, including the following:

1. Patients undergoing surgery, radiotherapy, chemotherapy, or hormone therapy for cancer
2. A range of cancer types: breast, gynecological, colorectal, and urological
3. Women and men
4. Patients aged older than 60 years and patients aged younger than 60 years

Patients’ eligibility for the study was assessed by cancer nurse specialists (CNSs) at the clinics. The inclusion criteria were as follows: patients were male or female; aged 18 years or above; receiving neoadjuvant, adjuvant, radical, or palliative treatments; at least 2 weeks after diagnosis (to allow patients enough time to come to terms with their diagnosis); able to give informed consent. The exclusion criteria were as follows: patients who do not have an estimated life expectancy of at least 12 months; patients who the clinician deems to be unsuitable for the research (eg, in a current state of crisis or have their own significant health or social problems, unable to provide informed consent, or other reason for not being approached about the study).

### Recruitment

A total of two CNSs assisted with recruitment by distributing information packs to eligible patients, containing an invitation letter, an information sheet, reply form, and prepaid envelope. The number of information packs distributed was recorded.

### Procedure

Interested participants contacted the lead researcher, and interviews were arranged in their own homes. The interview was confidential, and only the research group had access to the anonymized data. Participants consented to participate at the time of interview and completed a demographic questionnaire. Interviews were audio-recorded and transcribed verbatim.

### Interview Topic Guide

Relevant literature informed the development of a semistructured interview topic guide [20]. Topics included the following: information needs and information-seeking, communication with clinicians in consultations, experience with mobile technology, acceptability of an app intervention, desired app features, and the perceived benefits and disadvantages of, and barriers to, this type of intervention. All participants, regardless of characteristics such as cancer type and stages of disease, were asked the same questions. Participants who were unfamiliar with apps received a brief explanation by showing examples of existing apps on a smartphone (eg, notes app, social media apps, and email app). Additionally, features of the National Coalition for Cancer Survivorship “Pocket Cancer Care Guide” app were shown to provide these participants with examples of the types of features that could be used by patients with cancer. Participants who were familiar with apps were simply told that an app could help with a wide range of things, such as their information needs, communication with clinicians in consultations, adherence to medication, and social support. See Multimedia Appendix 1 for the topic guide.

### Analysis

Patients were interviewed until the research team felt that data saturation was reached. Data saturation was considered to have occurred when no new themes were identified for at least 3 interviews. Data were managed using the qualitative analysis software package NVivo 10 (QSR International). Interview transcripts were analyzed using a thematic analysis as this helps to provide insights by moving from a broad reading of the data to reporting patterns and themes, followed by their interpretation [26]. The analysis was neither considered purely inductive nor deductive. Instead, it can be considered as a blend of both approaches. Inductive approaches are those that start from the data and search the data for patterns that suggest general laws, ultimately aiming for generation of theories. In contrast, deductive approaches start with hypotheses that are derived from a theory, which are then tested against a body of data that was gathered to test the hypotheses. However, in practice, all research incorporates elements of both inductive and deductive logic [27]. Each transcript was read several times for familiarity, actively searching for, and noting, meanings and patterns. Initial codes were generated from each data item, and mind maps were created to identify the links between codes and possible overarching themes. Codes were then organized into meaningful subthemes and main overarching themes. Themes were reviewed and refined by reviewing each data item within a theme to ensure coherence. A total of 5 transcripts were independently analyzed by a second author to reduce the potential bias of subjectivity associated with coding and facilitate the interpretation of findings. Discrepancies were resolved through discussion.

The author, RR, a research associate and health psychologist, conducted and analyzed the data. Coauthor, FW, a senior lecturer and medical sociologist, double-coded a subset of transcripts as described above. Both authors maintained an awareness of how their own personal characteristics and values may have influenced data collection or analysis. For example, neither RR nor FW has had a previous diagnosis of cancer and therefore may not fully understand participants’ experiences or the psychosocial context. During interviews, some participants might have been wary of RR because of her profession as an academic researcher, and some participants may have assumed that she was in contact with their cancer clinician. RR was aware of how this might have influenced participants’ trust and openness during the interviews and so made every effort to build a rapport and trust before the interview to make the participants feel comfortable and at ease. RR assured participants that the interviews were confidential and that their views and opinions would not be discussed with their clinicians or affect their care in any way.

## Results

A total of 23 interviews were conducted between November 2014 and February 2015. The average length of the interviews was 43 min (range: 16-75 min).

### Sample Characteristics

A total of 130 information packs were distributed to eligible patients (40 urological cancer patients, 30 colorectal cancer patients, 30 gynecological cancer patients, and 30 breast cancer patients). Of these, 33 patients returned a reply form indicating interest in participating, of which 23 participated and completed the study (overall response rate: 18%). Of the responding patients, 4 did not answer the telephone or respond to emails, 4 stated that they were not feeling well enough to participate, 1 patient was on holiday, and 1 patient declined. Sample characteristics are presented in Table 1. The most common age category was 56 to 65 years (35%, 8/23) and the most common cancer type was colorectal cancer (44%, 10/23). The response rate from each cancer type was 7% (3/40) for urological cancer, 13% (4/30) for breast cancer, 16% (5/30) for gynecological cancer, and 33% (10/30) for colorectal cancer. The most common time since diagnosis was 1 to 2 years (35%, 8/23). Nearly three-quarters of participants were educated to at least the secondary level (74%, 17/23), and over a quarter were educated to the degree level (26%, 6/23). All participants were white Caucasian. In total, 74% of participants (17/23) reported that they owned (or co-owned) a smart device (smartphone or tablet).

**Table 1:** Sample characteristics.

Identification number	Age (years)	Gender	Cancer type	Time since diagnosis	Education level
P^a^1	66-75	Male	Urological	2-4 years	GCSE^b^/O levels^c^
P2	18-25	Female	Gynecological	3-6 months	Diploma
P3	66-75	Male	Colorectal	1-2 years	No education
P4	66-75	Male	Colorectal	2-4 years	Degree
P5	56-65	Female	Breast	3-6 months	Postgraduate degree
P6	66-75	Male	Colorectal	3-6 months	No education
P7	56-65	Female	Colorectal	2-4 years	Degree
P8	56-65	Female	Gynecological	5 years +	Degree
P9	85+	Female	Colorectal	1-2 years	No education
P10	66-75	Female	Other	1-2 years	No education
P11	46-55	Female	Gynecological	2-4 years	GCSE/O levels
P12	46-55	Female	Breast	1-2 years	Missing data
P13	56-65	Female	Gynecological	2-4 years	Degree
P14	66-75	Male	Colorectal	1-2 years	Postgraduate degree
P15	76-85	Male	Urological	1-2 years	Diploma
P16	56-65	Male	Colorectal	6 months-1 year	No education
P17	46-55	Male	Colorectal	6 months-1 year	Degree
P18	56-65	Male	Colorectal	2-4 years	GCSE/O levels
P19	36-45	Female	Breast	1-2 years	NVQ^d^/HNC^e^/HND^f^
P20	56-65	Female	Gynecological	1-2 years	Degree
P21	66-75	Female	Breast	5 years +	GCSE/ O levels
P22	76-85	Male	Colorectal	2-4 years	No education
P23	56-65	Male	Urological	3-6 months	GCSE/O Levels

^a^P: patient.

^b^GCSE: General Certificate of Secondary Education.

^c^O level: General Certificate of Education Ordinary Level.

^d^NVQ: National Vocational Qualification.

^e^HNC: Higher National Certificate.

^f^HND: Higher National Diploma.

### Interview Themes

From the interviews, 4 key themes were identified: (1) suggested app features, (2) anticipated benefits of app use, (3) potential disadvantages of app use, and (4) anticipated barriers to app use.

#### Theme 1: Suggested App Features

Most participants wanted informational features that would support self-management of their condition such as providing information on treatment-related side effects, cancer support services, lifestyle changes (eg, diet, exercise, and smoking), survival and recurrence rates, alternative therapies, managing finances, psychological support, and logistical issues:

Well the sort of information that I wanted was um symptoms, you’ve been told you’ve got colon cancer, like myself, um the sort of thing I wanted to find out was how curable is it? Um...treatments, I wanted to know what sort of treatment I was having.

P11; 46-55 years, gynecological cancer

Some participants suggested to include links to credible cancer information websites, as they reported that they found it difficult to navigate the internet and identify reliable information:

It [the app] could be used to direct them [patients] towards websites that contain that information, so you could use it as a roadmap, that I suspect could be useful.

P14; 66-75 years, colorectal cancer

Some participants suggested that a treatment-related symptom diary feature would be useful. Participants described how keeping a diary helped to predict how they would feel at certain times during their treatment and helped them to plan ahead to prevent or remedy symptoms and organize their diet and social calendar. Participants explained that they felt reassured by knowing when symptoms were likely to occur:

Uh like I told you when you start chemo, it’s really good for you to have a report, a detailed report of symptoms, how you feel. So throughout the cycles, not only for yourself to prepare yourself for what’s coming as well, for the nurses because they ask you, they ask you at every clinic, “How are you feeling?”, “How did it go?”. If you don’t write it down I can tell you, you will forget. If the app has um a way so that you could personalize your own link and then you can actually have a diary.

P19; 36-45 years, breast cancer

Many participants also suggested app features that would facilitate information exchange in consultations, such as a question prompt list (QPL; a tailored list of questions to be asked). It was anticipated that a QPL would be the most useful feature for patients:

I think if the app does that, you know gives you a list of questions that would be useful for you to ask so you can write them down...I think it’s extremely useful because at least you’ve got your mind set to ask the questions if you’ve got any...If I had any questions when I got home, I’d have to ring back and say, “Look I don’t understand this,” you know so I think if the app does that, that’s really good.

P19; 36-45 years, breast cancer

Participants also suggested including a glossary that provided definitions of cancer terms, as they recalled not being able to understand the terminology used by clinicians or that in information resources such as leaflets:

I think that would be useful and explain what that means to them, because I think an awful lot of people would be to a degree, a little bit in awe of what the doctor is saying because they’re not trained as uh doctors they wouldn’t understand completely, and maybe there’s a slight reluctance to say to the doctor, “Well explain that more fully, be more open with me” because of that if you like, power differential, between the patient and the doctor, so having the ability to go to an app afterwards, provided you remember what all the big words were.

P14; 66-75 years, colorectal cancer

Participants also suggested app features that would raise awareness of, and increase patients’ access to, other types of support. Some participants suggested including links to local cancer support services, such as psychological support services, and help with managing finances and cancer charity websites. It was hoped that this would raise awareness of and increase access to some of the services that participants found helpful during their illness (eg, a financial benefits advisor for patients who were unable to work):

I think there’s a lot of things the app could help to link up a bit. You know, there is a lot of stuff [cancer services] out there, putting it in one place would probably, I would’ve thought would be helpful, so that people haven’t got...I kind of stumbled across things by accident, like I didn’t realise that Macmillan did the complimentary therapy services thing, but there’s also links to sort of art therapy and all sorts of things that are there, and they’re there for families as well and I didn’t know they were there initially.

P8; 56-65 years, gynecological cancer

Many participants recalled how they exchanged information with other patients with cancer, both face-to-face at hospitals and on the internet (eg, information on remedies for treatment side effects), which they described as invaluable. Therefore, some participants suggested including a social feature or links to existing social forums or media. Participants also suggested to include links to information on the internet or local support groups to meet other patients, as they found it difficult to locate information on these services:

Um, I think support side of it is very important, to give the information about support, um support groups, cos that’s what I couldn’t find, I couldn’t find any support groups. It was only about last year I found a support group near home.

P11; 46-55 years, gynecological cancer

#### Theme 2: Anticipated Benefits of App Use

Participants identified the potential benefits of an app that would help patients meet their information needs. Owing to increased access to reliable and relevant information, participants anticipated that future patients would have a better understanding of their condition and the information provided in consultations. A more informed patient was expected to be able to identify the side effects of treatment and treat them accordingly, which may prevent complications and potentially improve their quality of life. For example, one patient reported that her lack of knowledge on the importance of monitoring her temperature during treatment almost led to her hospitalization:

On the Sunday night when I was feeling like death I took my temperature and it was 37.5 so I took it with another thermometer and it was 37.4, because 37.5 is the magic number [the threshold] I went to bed, in the morning I took my temperature and it was 37.9 ...but because I didn’t want to go in, “Its 37.4, I don’t want to go in”. If I had understood the very important aspect of that, I would have gone in to hospital that night.

P5; 56-65 years, breast cancer

A more informed patient was also expected to have lower levels of anxiety throughout their illness owing to having a better understanding and more realistic expectations of their prognosis and treatment:

I think uh, give information on treatment, be specific about what’s involved with chemotherapy because people are afraid of chemotherapy and if it was explained to them beforehand they might not be as afraid. Explain about what happens with radiotherapy, as again, people are afraid of it.

P7; 56-65 years, colorectal cancer

There was some evidence to suggest that an app could help patients increase their confidence in actively participating in their care and to communicate with their clinicians:

What benefits do you think there might be for patients using this type of app?

Interviewer

Well it might help them, it might help them get some confidence, within the system, because if they, you know if it opens up questions and answers session when they go [to clinic] it’s going to make them more confident next time isn’t it? It’s going to help encourage their relationship with their practitioners, so you know with their doctors, so...

P20; 56-65 years, gynecological cancer

Participants also highlighted the benefits of smart technology. Accessing cancer-related information and resources via an app was expected to be less burdensome than searching through printed leaflets and booklets:

You have these pile of booklets off them and when you see all that you’re like, “Oh have I really got to read all that?”, so if you’ve got an app there it’s easier then isn’t it? It’s like you haven’t got to carry everything around, and say you’re in an appointment, you can just pull the app up on your phone and just read up on it, rather than carrying all these massive books with you.

P2; 18-25 years, gynecological cancer

#### Theme 3: Potential Disadvantages of App Use

Few participants were concerned that some patients might become anxious if they misinterpreted information or were misinformed by inaccurate information:

One point that might manifest itself would be Joe Bloggs getting the wrong end of the stick, when they’ve been diagnosed with a particular condition, their research may take them away from the condition to something else, and maybe anxiety could set in as a result of that, because they’ve over researched it perhaps and frighten themselves.

P14; 66-75 years, colorectal cancer

Additionally, a small number of participants also worried that if patients actively used an app in a consultation, it might distract them from the conversation with the clinician:

In my case it [the app] would hinder communication...because you’re looking at this [the app] and you’re not looking at them [the clinician] and you’re just reading a list.

P10; 66-75 years, other cancer

#### Theme 4: Anticipated Barriers to App Use

Most participants reported that they did not foresee any barriers to the use of an app. The most commonly anticipated barrier was patients’ age and experience with smart technology. Some participants anticipated that many older patients would lack the knowledge and experience to be able to use an app, in comparison with younger patients:

If you said to me there’s this app called such and such then I’d just go and look at it and find it out for myself, like my dad bless him who’s 82 and he plays around with his laptop um he wouldn’t know like to look at the little words and to click on them and things and explore an app you know? ...When somebody of your generation finds it, oh that sounds patronizing but imagine that um you know, there are some people they still don’t know what an app is.

P13; 56-65 years, gynecological cancer

In support of these views, few participants anticipated that they would be unlikely to use an app as they preferred traditional methods to gather information, such as asking a nurse or friend. Some participants also anticipated that a minority of patients would favor an avoidant coping approach and only want minimal information to minimize their anxiety:

Do you think if you had a Smartphone or tablet that you would use, or try to learn to use the app?

Interviewer

Probably not, I would probably still ring the nurses.

P10; 66-75 years, other cancer

I can’t have enough information, but I know from my experience people don’t want a lot of information.

P5; 56-65 years, breast cancer

Access to smart devices, in terms of cost or access to the internet, was also highlighted as a barrier by participants, though patients who did not own a smart device often had access to one via family or friends:

...The only barrier I can think of is that some people do not have any access to the Internet and I suppose that’s something that you just have to accept, you know that’s not a reason for not producing something, but that’s the only barrier that I can see, in that people, there are people who don’t have Internet access.

P13; 56-65 years, gynecological cancer

Few participants were concerned about the accuracy of information sourced from an app; however, they suggested that future patients would be likely to trust an app if it was endorsed by their clinicians or affiliated with a reputable cancer charity. Similarly, some participants who were less familiar with smart technology were concerned about the confidentiality and security of personal information:

How reliable it is? For example, if you told me that the app had support or background from the cancer research, I would be more than happy to you know to look up anything that I would read, or that I would obtain from the app was accurate and that I could rely on, for me that would be “the” thing reliability, where it comes from, what’s the basis, can I trust it personally?

P19; 36-45 years, breast cancer

...As long as it keeps confidentiality, which is I think absolutely imperative, I mean certain things slip past the old uh marker at times, um, yeah I think that’s generally that’s the most important thing confidentiality is not in any way breached, you know.

P3; 66-75 years, colorectal cancer

## Discussion

### Principal Findings

This is the first study, as far as we are aware, to explore the views of patients with cancer about an app that aims to help them meet their illness-related information needs in noninpatient settings. The primary aim of this study was to explore the value of an app for patients and to establish the type of app required and its potential outcomes. Suggestions for app features indicated the need for an app that supports patients to retrieve the information that they need from their short time in consultations, facilitates understanding, collates large amounts of information regarding available services, and helps patients to navigate through them. The potential benefits of this type of app included a more informed patient, improved quality of life, reduced anxiety, and increased confidence to participate in their care. The benefits appeared to outweigh the potential disadvantages, which were identified as increased anxiety and distraction in consultations. The anticipated barriers to app use included age and experience with smart technology, access to smart devices and the internet, an avoidant coping approach, and security and confidentiality of personal information.

Patients’ desires for particular app features reflected their experiences of information gathering and understanding during and between consultations. First, participants suggested app features that would facilitate patients’ self-management of their condition by providing detailed information on their condition. This type of information might also prevent hospitalizations [28]. Participants also suggested links to reliable websites to help them navigate the internet and source accurate information. As the internet is now a common health information resource, studies have highlighted the importance of guiding patients and educating them on how to filter accurate health information [29,30]. However, information needs vary throughout a patient’s illness, as well as among patients with different types of cancers (eg, common vs rare cancers) and stages of a disease (eg, stage I vs stage IV). Although it would not be feasible to develop an app that includes all the possible information to fit all patients for all types and stages of cancer, the addition of a QPL, in which patients could add their own specific questions to ask their clinician during consultations, would enable an app to facilitate more personalized care that meets the individual needs of each patient.

Second, patients suggested app features to enable them to overcome barriers to communication in consultations. For example, a QPL might help patients to remember to ask important questions. Reviews of the use of paper-based QPLs for cancer consultations have suggested small but positive effects on communication, question asking, and information recall [31,32]. A glossary of cancer terms was also suggested in the hope of enabling patients to develop a better understanding of cancer-related information.

Finally, patients highlighted other negative consequences of a cancer diagnosis, such as financial and psychological issues, and suggested that an app could provide information on the available cancer services for patients to raise awareness of and signpost them to relevant support. Similarly, patients suggested to include a feature than enables contact with other patients for emotional support. This finding is consistent with previous studies on the benefits of social support during cancer [33].

The most commonly anticipated outcome of this type of intervention was a more informed patient, which, in turn, was expected to lead to a range of other benefits, such as increased quality of life, reduced anxiety, and increased confidence to participate in their care. Previous studies have provided evidence for these types of benefits because of improved communication with clinicians in consultations and increased access to information outside of consultations [34-36]. Suggestions that this type of app might increase users’ knowledge of their condition, and participation in consultations, might indicate that this type of app has the potential to increase patients’ levels of activation [37].

Some participants in this study expected that older patients would be less likely to use an app, and consistent with these expectations, few older patients in the study thought they would not use an app, and instead preferred more traditional methods of information gathering. In contrast to these expectations, studies show that many older patients are willing to learn to use new technology if they think that it will benefit them [38,39]. Other perceived barriers to app use included access to smart devices, the perceived reliability and security of information, and an avoidant coping approach. Access to smart devices is likely to be a temporary barrier, as ownership of smart devices is increasing rapidly in the United Kingdom across all demographic groups [40]. Furthermore, affiliation with a reputable organization and development of an app that does not require personal information will reduce concerns about reliability and security of information. A minority of participants appeared to have an avoidant coping style and therefore anticipated that they would not want to learn more about their illness. However, other features, such as links to information on psychological support, might still be of use for this group of patients.

Finally, two potential disadvantages of using this type of app were suggested including increased patient anxiety and distraction in consultations, potentially leading to poorer communication with clinicians. The risks of these potential consequences may be minimized by including only reputable information resources and avoiding active engagement with the app during communication in consultations (ie, use as a reference and not to type notes). Overall, the anticipated benefits of this type of intervention appeared to outweigh the potential disadvantages.

### Implications

This study presented novel findings on the preferences of patients with cancer regarding the development of an app to help meet illness-related information needs, including the potential outcomes and benefits of this type of intervention. These findings can be used to develop intervention objectives and inform the selection of app features [21]. For example, based on patients’ views reported in this study, the objectives of the intervention might be to facilitate the development of patients’ understanding and self-management of their condition, and it is anticipated that this could be achieved by including the following app features that enable patients to (1) gather, exchange, and understand information during consultations with their clinicians, (2) access and navigate reliable cancer information resources on the internet, and (3) identify and access patient services that will provide further information and support where needed (such as social support). This study also identified the potential disadvantages of, and barriers to, this type of an app, and these findings can be considered during app design to optimize its uptake, usability, and usefulness [21]. For example, patients suggested that lack of access to the internet might prohibit app use for some, and so a design objective could be to design an app that may be used offline, without an internet connection. Similarly, to circumvent some patients’ worries about the security and confidentiality of the app, a further design objective could be to design an app that does not require the input of identifiable or personal information, such as names and addresses.

### Limitations

The varied sample of patients is a strength of this study; however, there are several limitations to consider. The study had a low response rate, included high numbers of smart technology owners, and most participants had higher educational levels. Additionally, information on the key characteristics of those who declined to participate was not collected, and the different cancer sites had varying response rates. The lowest response rate was from patients with urological cancer. Owing to these limitations, the sample may have included those with more favorable perceptions of an app. Those who declined to participate may have not been familiar with smart technology or disliked the idea of an app for patients with cancer. Similarly, patients who do not own smart technology were likely to be less familiar with it and may therefore have had more negative perceptions about this technology. The low response rate from patients with urological cancer and a fairly low response rate from patients with breast and gynecological cancers might indicate differences in opinion between cancer sites. For example, patients with urological cancers are more likely to be older male patients, and studies have shown that this population group is less likely to engage in health information-seeking and other related Web-based activities [41].

A further limitation of this study is that all participants were white Caucasian. Although some studies suggest little evidence for a digital divide by race or ethnicity [41], some report that black and ethnic minority groups, such as African American and Chinese, may have different experiences of cancer and use different information sources, and may therefore have different needs and preferences regarding an app [42,43].

Providing examples of types of app features that could be used by patients with cancer before beginning the interview might have influenced some participants’ responses owing to social desirability. The risk of this bias was minimized as the interviewer explained that all opinions were valued, both positive and negative, to develop an app that would be most useful for future patients.

Finally, participants were asked to reflect on a hypothetical scenario where an app could be available for them to use during their illness. Participants were also asked to anticipate *potential* benefits and disadvantages of, and barriers to, a hypothetical app. As a result, the data are not necessarily grounded in concrete experiences and therefore may not translate into engagement.

### Conclusions

This study is the first to explore the preferences of patients with cancer regarding an app that aims to help them meet their illness-related information needs in noninpatient settings. Participants highlighted types of app features that they would find useful, specifically an app that would enable patients to develop an understanding of and subsequently self-manage their condition, including features to support information exchange and understanding in consultations and features to increase access to support for patients. The potential outcomes of this type of an intervention were highlighted, and the benefits of an app appeared to outweigh the few possible disadvantages and barriers to app use.

## Appendix

Multimedia Appendix 1 Topic guide.

## Abbreviations

CNS: cancer nurse specialist
NHS: National Health Service
QPL: question prompt list
