# Supplementary material for: The Preferences of Patients With Cancer Regarding Apps to Help Meet Their Illness-Related Information Needs: Qualitative Interview Study
Source: JMIR Mhealth Uhealth. 2019 Jul 31;7(7):e14187. doi: 10.2196/14187 (PMC6693303; doi:10.2196/14187)
Supplement: Multimedia Appendix 1 [file mhealth_v7i7e14187_app1.pdf]

Exploring the potential of a smartphone 'app' for cancer patients

Patient Interview Schedule

1. Introduce myself, explain where I am from, ensure they're comfortable etc.

2. Check understanding of reason for meeting, give an opportunity for questions:

"Before we start, I wonder if you have any questions about this study or about why I've come to talk with you today?"

3. Set the focus of the interview. Base this around the following script:

"Thank you for agreeing to take part in this study. I want to understand what patients with cancer think of a potential smartphone/tablet app for patients with cancer. The interview will take around half hour/forty minutes".

4. Establish whether participants understand what an app is and whether they are familiar with them:

"Are you familiar with apps? Do you know what an app is?"

5. If the participant is familiar with app, continue with the following:

"This app would be for use *after* diagnosis and could potentially help patients with a wide range of things. For example, it could help with their information needs or help communication with clinicians in consultations. The app could also help with things like adherence to medication or social support and so on".

5a. If the participant is not familiar with apps, continue with the following:

Show apps on smartphone to participant (e.g. the notes app, Facebook app, email app) to explain what an app is. "**I will show you an example of an app for patients with cancer that is already available, just give you an idea of such an app".** Brief demonstration of NCCS Pocket Cancer Care Guide here.

6. "At the end of this study, we aim to develop an app based on the information gathered from interviews with clinicians, patients with cancer and their relatives. I am interested in your views on a potential smartphone/tablet app for patients with cancer. There are no right or wrong answers to my questions, I am interested in what *you* think."

7. After establishing what is understood about the study, and answering any questions, explain that the interview will be recorded:

"I would like to record what you say as that saves me having to write when you're talking and means that I can concentrate on what you're saying. The recording will only be heard

by people who are working on this project. The interview will be transcribed and your identity and the identity of any person you talk about today will be anonymised in any published work. The interview is completely confidential and no information will be relayed back to your doctor. This will interview, and your views and opinions, will not affect your care in any way. Is that okay with you?”

8. Go through the information sheet again and consent form. Obtain consent for the interview and for the recording. If not already done, set up and switch on the recording equipment while the participant signs the consent form.

Opening question

- Can you tell me why you were interested in taking part in this study?

Information needs

- Do you like to have information about your illness? (prompt: why, how much information?)
- Where do you normally get information about your illness (prompts: health professionals, friends/family, book/internet etc)?
- What sources do you use? Why?
- Was there a particular point where you felt like you needed more information?

Communication with clinicians in consultations, part 1

- How do you find talking to clinicians about your illness?
- How do you feel when you ask the doctors and nurses questions about your illness? (prompts: is it easy or difficult, what makes it hard to ask them questions?
- What makes it easier to ask doctors and nurses questions?
- Were there any barriers to communicating with your doctors/nurses?
- How do you remember all the information about your illness?
- Do you find it easy to talk to your family/friends about your illness?

Experience with mobile technology

- Do you have experience of using a mobile/smartphone/ tablet computer?
- What was your experience of using a mobile/ smartphone/tablet computer? OR Why don’t you use a mobile/smartphone/tablet computer?
- What do you use your mobile/smartphone/tablet computer for?
- Are you familiar with ‘apps’?
- Have you ever, or do you currently use an app to help with your health or cancer? Why?

Perceived acceptability of an app

- Do you think you would find an app acceptable to use? (i.e. easy or difficult to use?)
- Do you think other patients who have cancer would find an app acceptable to use?
- Do you think families or friends of patients would want to use an app? Why?
- Do you think you/other patients would need initial training to use an app?
- If the app was made available, would you download it and use it?
- Would you have any concerns about using an app?

Desired app features

- What things would you like the app to do? Why?
- What sort of things do you think family and friends of patients would want the app to do?
- Is there anything that you would not want the app to do? If so, why?

Perceived benefits of the app

- What benefits do you think there might be for patients using the app?
- What benefits do you think there might be for clinicians?
- What benefits do you think there might be for the family/friends?

Communication part 2

- Do you think the app would have an impact on the way you talk to your doctors and nurses about your illness? How? Could it help? Could it make it more difficult to talk to them?
- Do you think the app might affect the way you talk to your family and friends? In what ways?

Perceived barriers of the app

- Do you think there may be problems with using the app? What do you think the problems might be of using the app?

Patient type

- Are there any particular types of patient that you think might find the app most useful? (prompt: age groups, patients at different stages of diseases). Why?
- When do you think it might be most useful for patients to use the app? (i.e. after diagnosis, after the dust has settled, pre-treatment, post-treatment and discharge)

- If any of your friends had cancer would you recommend they use it? Do you think they would use it? Why/why not?

Final question

- What was your overall experience of being involved in this study?
